# Transmaxillary approach to the cranial base: an evaluation of 11 cases

**DOI:** 10.1016/S1808-8694(15)31372-0

**Published:** 2015-10-17

**Authors:** Antonio Figueiredo Caubi, Carlos Augusto Pereira Lago, Belmiro Cavalcanti do Egito Vasconcelos, Emanuel Dias e Oliveira Silva, Nelson Studart Rocha, Hécio Henrique Araújo de Morais

**Affiliations:** 1 Specialist and master’s degrees in Buccomaxillofacial Surgery and Traumatology / doctoral student in Buccomaxillofacial Surgery and Traumatology, FOP/UPE. Adjunct professor, Faculdade de Odontologia de Pernambuco, FOP/UPE; 2 Specialist and master’s degrees in Buccomaxillofacial Surgery and Traumatology / doctoral student in Buccomaxillofacial Surgery and Traumatology, FOP/UPE. Adjunct professor, Faculdade de Odontologia de Pernambuco, FOP/UPE; 3 Specialist, master’s and doctoral degrees in Buccomaxillofacial Surgery and Traumatology. Adjunct professor of Buccomaxillofacial Surgery and Traumatology, Faculdade de Odontologia de Pernambuco, FOP/UPE. Coordinator of the master’s and doctoral programs in Buccomaxillofacial Surgery and Traumatology, FOP/UPE; 4 Specialist in Buccomaxillofacial Surgery and Traumatology, regent of the Buccomaxillofacial Surgery and Traumatology Discipline, FOP/UPE; 5 Specialist in Buccomaxillofacial Surgery and Traumatology, buccomaxillofacial surgeon, Hospital Getúlio Vargas - PE; 6 Specialist in Buccomaxillofacial Surgery and Traumatology, buccomaxillofacial surgeon, Hospital da Restauração. Faculdade de Odontologia de Pernambuco - FOP/UPE. Buccomaxillofacial Surgery and Traumatology Discipline

**Keywords:** maxilla, osteotomy

## Abstract

Surgical access to the skull base is always difficult, especially because of the noble anatomic structures present there. Maxillary osteotomy provides direct view to the clivus region and the neck spine, and it also bears less morbidity when compared to the many other accesses described in the literature.

**Aim:**

to assess 11 patients submitted to transmaxillary osteotomy, describing the surgical technique and postoperative results and complications.

**Materials and Methods:**

A retrospective study involving eleven patients submitted to transmaxillary approach to the brainstem. We studied dental occlusion, trans and postoperative bleeding, bone necrosis and soft tissue alterations. All followed the same surgical protocol and were followed up for two years.

**Results:**

after treatment, all the patients improved in their clinical status and had no neurological complication, trans and postoperative hemorrhage or major complications were seen. Among the complications, two patients had incomplete maxilla fracture, two had laceration of their nasal mucosa and one had, as late complication, an oral-sinusal fistula.

**Conclusion:**

Transmaxillary osteotomy provided proper access to the clivus for brainstem decompression with low rate of complications in this series.

## INTRODUCTION

Surgical access to disease of the cranial base has always been a treatment challenge, as this anatomical area is surrounded by structures that are essential for life.^1^, ^2^ The modified Le Fort I osteotomy has been employed for removing tumors in this area; it provides a frontal view of the clivus, no skin incisions are required, and there is less risk of vascular and nerve injury.^3^, ^4^

A frontal approach (craniotomy) or a lateral approach (facial bone osteotomy) involves excessive tissue retraction and an increased risk of vascular and nervous injury, which may result in permanent loss of facial motor function.^1^, ^5^

The purpose of our study was to present cases in which the transmaxillary approach to the cranial base was used, describing the surgical technique, its results and complications.

## MATERIAL AND METHOD

**Figure 1:**
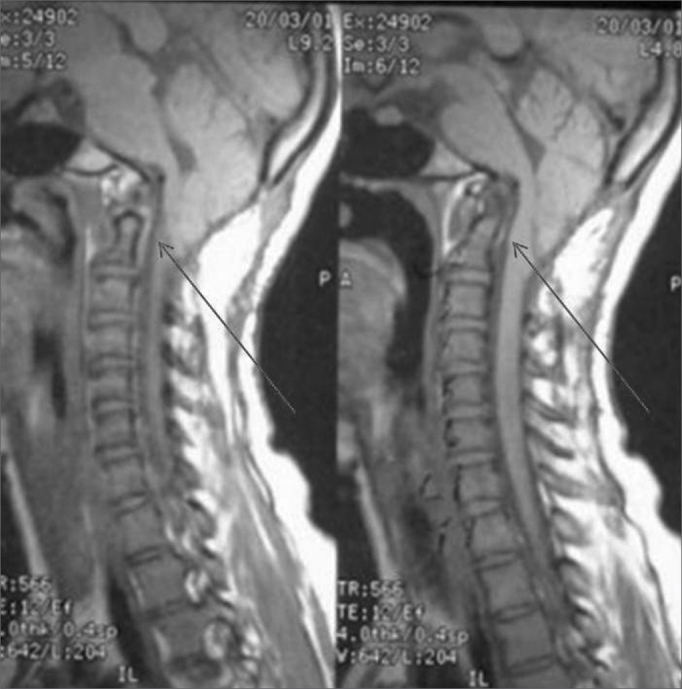
Magnetic resonance image showing brainstem compression by the odontoid process.

Eleven patients with a neurological condition resulting from basilar invagination and with brainstem compression by the odontoid apophysis, were subjected to a maxillary approach for accessing the clivus area (Fig. 1). The main symptoms were gait disorders and lower limb weakness progressing over two years (Table 1).

**Table 1:** Epidemiological findings of patients.

Nº	Gender	Age	Main Complaint	Diagnosis
1	Male	19	Gait disorder	Basilar invagination
2	Female	26	Lower limb weakness	Basilar invagination
3	Male	34	Gait disorder	Basilar invagination
4	Male	43	Gait disorder	Basilar invagination
5	Male	39	Lower limb weakness	Basilar invagination
6	Male	32	Gait disorder	Basilar invagination
7	Female	16	Gait disorder	Basilar invagination
8	Male	48	Sexual impotency	Basilar invagination
9	Male	39	Lower limb weakness	Basilar invagination
10	Male	31	Torticollis	Basilar invagination
11	Male	45	Lower limb weakness	Basilar invagination

Transmaxillary surgery was done by the buccomaxillofacial surgery and traumatology team. Surgical planning included conventional radiography (panoramic maxillary view and lateral cephalometry), computed tomography and magnetic resonance imaging of the skull and cervical spine.

The same surgical team performed all procedures; the surgical procedure is that described by Eisig et al.,^5^ as follows: a premolar to premolar vestibular incision was done on the maxilla; at this point the infraorbitary nerve was identified, the lateral maxillary wall, the piriform aperture and the zygomatic pillar of the maxilla should be exposed. A second vertical approach on the dental papillary area between the central incisors was done for division of both maxillae.

**Figure 2:**
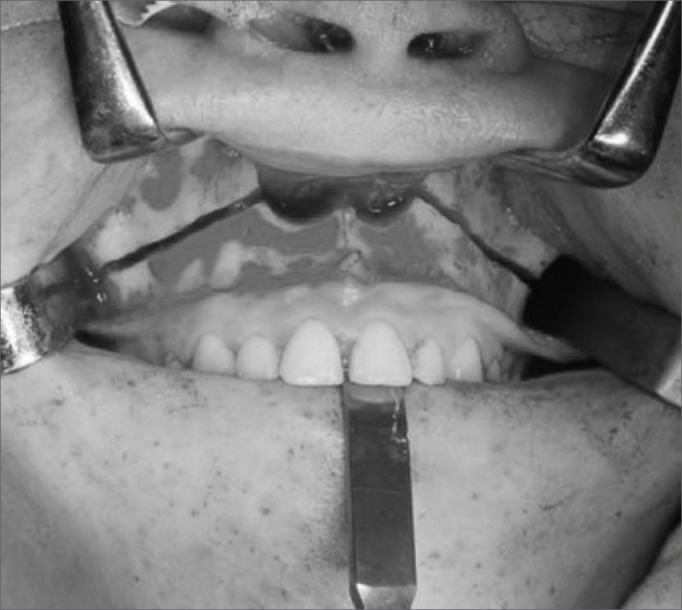
Maxillary Le Fort I osteotomy.

**Figure 3:**
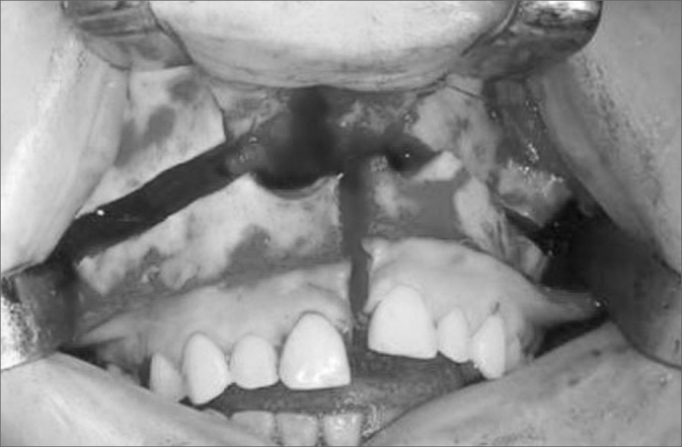
Vertical osteotomy leading to sagittal division of the maxilla.

Titanium miniplates were placed, prefolded and screw holes were perforated. A horizontal maxillary osteotomy was done using a rotating device starting anteriorly from the piriform aperture and canine pillar towards the lateral wall of the maxilla and the zygomatic pillar (Fig. 2). The nasal septum was separated from the maxillary septal crest using a guided chisel to avoid laceration of the nasal mucosa. Posterior osteotomy was done using curved chisels to separate the pterygoid process from the sphenoid on the anterior maxillary portion. A down-fracture was done and median osteotomy was performed from incisives to the hard palate (Fig. 3).

**Figure 4:**
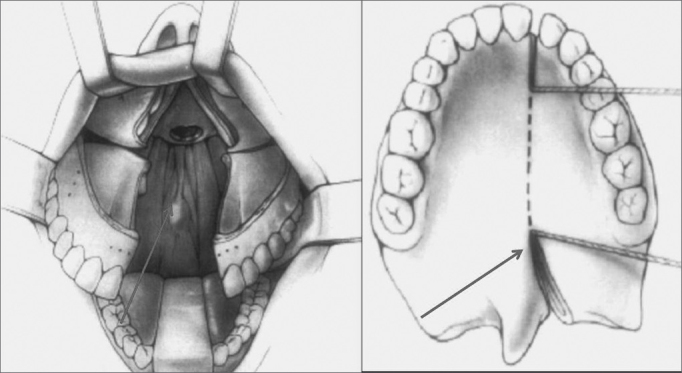
Representation of a hard and soft palate incision / maxillary division and placement of retractors.

A lateral incision to the uvula on the soft palate and a median incision along the hard palate to the gingival between the incisives were done. Along the soft palate area a full incision was done to expose the nasopharynx. Both palatine flaps were detached and the major palatine artery was preserved. The maxilla was divided into two segments and positioned laterally with a Coldman palate retractor (Fig. 4).

The neurosurgical team approached the clivus through an incision on the posterior pharyngeal wall; an odontoidectomy was done using a rotating device.

**Figure 5:**
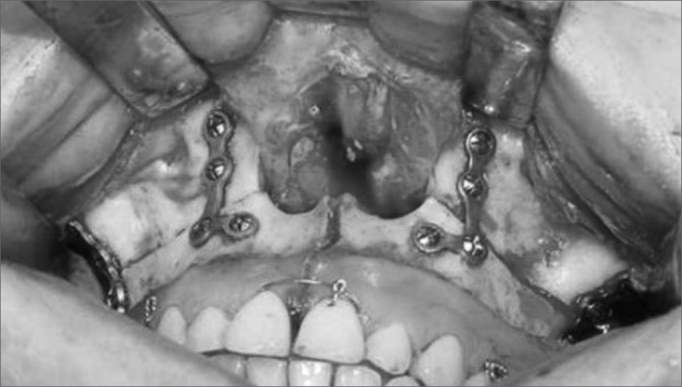
Maxillo-mandibulary block and internal rigid fixation.

At the end of the procedure and after the pharynx was closed, the maxilla was placed using a previously made acrylic resin surgical guide (with preoperative dental occlusion). The titanium miniplates were placed in the previously perforated areas (canine and zygomatic pillars) and on the floor of the piriform aperture (Fig. 5).

Layered Vycril (Ethicon®) sutures were used in the soft palate; a single layer of Vycril was used on the hard palate. The vestibular mucosa was sutured continuously using Vycril.

All patients were monitored postoperative for a mean period of two years. Pre- and postoperative dental occlusion, intra- and postoperative bleeding, surgical wound dehiscence, oronasal fistulae and bone necrosis were investigated.

The Research Ethics Committee of our institution judged and accepted this paper (protocol number CEP/ UPE: 038/08).

## RESULTS

Double-segmented maxillary osteotomy made it possible to approach the cranial base and the odontoid process with no complications or significant events in all cases.

Any sensory or motor deficit and intra- or postoperative bleeding were noted. No case developed infection, necrosis or bone sequestration. There were no postoperative meningitis and/or cerebrospinal fluid leaks.

Healing of the vestibular mucosa and soft palate were normal; one patient had wound dehiscence in the hard palate. This case progressed to a bucconasal leak that was treated surgically.

There were undesirable posterior maxillary fractures in two cases due to incomplete osteotomy in that area. Treatment consisted of completing the posterior osteotomy, which did not affect the end result.

Nasal mucosa laceration was seen in two other cases due to insufficient detachment of the nasal mucosa on the floor and lateral wall of the piriform aperture. The mucosa was sutured before repositioning and fixating the maxilla.

Regarding soft and hard palate incisions, there were no cases of velopharyngeal incompetence in this series. Postoperative dental occlusion was unaltered in all cases; no teeth lost their vitality.

## DISCUSSION

The Le Fort I osteotomy for approaching diseases in the cranial base is intimately linked to the history of the procedure first described by Cheever (apud Moloney and Worthington, 1981),6 in which maxillary osteotomy was used for removing a tumor from the nasopharyngeal area.

An intraoral access reduces the risk of injury to the 7th cranial nerve, and is more esthetic due to the absence of skin incisions. However, maxillary vascular injury due to an inappropriate surgical technique may result in maxillary necrosis.^3^, ^4^

The risk of vascular injury in these osteotomies has led many authors to question this procedure in the past decades. Such complications range from the loss of dental elements to alveolar segment or total maxillary necrosis. Bell7 described the biological basis for bone repair and revascularization following the Le Fort I osteotomy. In these studies, histological and microangiographic findings revealed that the buccal pedicle associated with palatine mucosa vasculature provided the blood supply needed for adequate tissue repair. In these transmaxillary surgical cases, access along the full extent of the palate and sagittal maxillary division were undertaken in addition to the conventional horizontal maxillary osteotomy; there were no cases of inadequate bone healing or infection. Good surgical technique is paramount, avoiding excessive pulling of the maxillary vascular pedicle or excess palatine mucosa detachment; these measures favor adequate postoperative vascularization even in procedures lasting over 10 hours, such as these.^8^, ^9^

Intraoperative hemorrhage may result from excessive stretching of the descending palatine artery or injury to the pterygoid plexus during disjunction of the pterygomaxillary process, especially if the osteotome is placed incorrectly or if excessive force is applied during osteotomy. Some authors have suggested that the descending palatine artery should be ligated to minimize the risk of intraoperative bleeding.^10^, ^11^ Our approach was to preserve the vascular bundle, restricting ligature to selected cases where the maxilla cannot be mobilized without this measure.

Williams et al.^12^ presented their experience in seven cases of transmaxillary surgery; one case developed meningitis associated with a cerebrospinal fluid leak and was treated with antibiotics. In the same study, postoperative dental malocclusion was noted in three cases; additionally, one patient developed velopharyngeal incompetence. Still another patient required incision and drainage of a soft tissue abscess.

Eisig et al.^5^ presented a review of nine cases of transmaxillary osteotomy for the removal of tumors in the clivus. In this study complications included two cases of cerebrospinal fluid leaks with meningitis, treated with antibiotics during 21 days. Another patient lost two teeth and part of the alveolar bone. Rehabilitation of that are required a further procedure for placing dental implants. A fourth patient developed an internal carotid artery pseudoaneurysm that required surgery.

There was a postoperative bucconasal leak in our series due to palatine suture dehiscence. Williams et al.^12^ recommends not suturing that area to minimize the risk of leaks in that author’s opinion. Eisig et al.^5^ routinely sutures the palate and has reported no case of postoperative leaks in that area.

The median area in which vertical osteotomy is done is anatomically the point of maximum bone resistance; the nasal septum is found along this area and there is less palatine mucosa in this region,^13^ which explains why there may be postoperative leaks. Our approach is to close the incision site using slow resorption and resistant sutures.

Suturing the soft palate by layers is essential to minimize the risk of postoperative velopharyngeal incompetence.^14^ There were no cases of cerebrospinal fluid leaks or meningitis in our 11 cases; this was probably due to a restricted access to the brainstem and odontoid process of the second cervical vertebra, which differs from other authors who use more ample surgical access that communicates the cranial cavity with the external environment.

Surgical results were not compromised in the two cases in which undesirable fractures occurred due to incomplete osteotomies. Full exposure and detachment of the posterior maxillary area are important measures to visualize and correctly divide the perpendicular plate of the palatine bone and the pterygomaxillary suture.

A surgical guide - made beforehand with cast models to reproduce the patient’s preoperative dental occlusion - minimized the possibility of postoperative dental malocclusion. This measure was important for avoiding postoperative dental disorders in our series.

## CONCLUSION

Transmaxillary osteotomy provided ample frontal access to the cranial base at the same time avoiding motor deficits and esthetic changes, with a low complication rate in our series.
